# Building capacity for integrated knowledge translation: a description of what we can learn from trainees’ experiences during the COVID-19 pandemic

**DOI:** 10.1186/s12961-022-00900-8

**Published:** 2022-09-15

**Authors:** Priscilla Medeiros, Celia Laur, Tram Nguyen, Meghan Gilfoyle, Aislinn Conway, Emily Giroux, Femke Hoekstra, Jean Michelle Legasto, Emily Ramage, Brenda Tittlemier, Brianne Wood, Sandy Steinwender, IKT Research Network Trainee Group, Cheryl Moser, Nicole MacKenzie, Ilja Ormel, Charly Degen

**Affiliations:** 1 Integrated Knowledge Translation Research Network Trainee Group, Ottawa, ON Canada; 2 Women’s College Hospital Institute for Health System Solutions and Virtual Care (WIHV), Toronto, ON Canada; 3 Women’s College Research Institute, Women’s College Hospital, 76 Grenville Street, Toronto, ON M5G 1N8 Canada; 4 Clinical Epidemiology Program, Ottawa Hospital Research Institute, Ottawa, Canada; 5 School of Epidemiology and Public Health, University of Ottawa, Ottawa, Canada; 6 School of Medicine, University of Limerick, Limerick, Ireland; 7 Better Outcomes & Registry Network (BORN) Ontario, Children’s Hospital of Eastern Ontario, Ottawa Children’s Treatment Centre, Ottawa, ON Canada; 8 Children’s Hospital of Eastern Ontario Research Institute, Children’s Hospital of Eastern Ontario, Ottawa, ON Canada; 9 Faculty of Medicine, University of Ottawa, Ottawa, ON Canada; 10 School of Health and Exercise Sciences, University of British Columbia, Kelowna, BC Canada; 11 Temerty Faculty of Medicine, Rehabilitation Sciences Institute, University of Toronto, Toronto, ON Canada; 12 Department of Physical Therapy, Temerty Faculty of Medicine, University of Toronto, Toronto, ON Canada; 13 School of Health Sciences and Priority Research Centre for Stroke and Brain Injury, University of Newcastle, Callaghan, NSW Australia; 14 Applied Health Sciences, University of Manitoba, Winnipeg, MB Canada; 15 Medical Education Research Lab in the North (MERLIN), Northern Ontario School of Medicine, Thunder Bay, ON Canada; 16 Health Information Science, Faculty of Health Sciences, Western University, London, ON Canada

**Keywords:** Integrated knowledge translation, Trainee, COVID-19, Co-production

## Abstract

The use of collaborative health research approaches, such as integrated knowledge translation (IKT), was challenged during the COVID-19 pandemic due to physical distancing measures and transition to virtual platforms. As IKT trainees (i.e. graduate students, postdoctoral scholars) within the Integrated Knowledge Translation Research Network (IKTRN), we experienced several changes and adaptations to our daily routine, work and research environments due to the rapid transition to virtual platforms. While there was an increased capacity to communicate at local, national and international levels, gaps in equitable access to training and partnership opportunities at universities and organizations have emerged. This essay explores the experiences and reflections of 16 IKTRN trainees during the first 2 years of the COVID-19 pandemic at the micro (individual), meso (organizational) and macro (system) levels. The micro level, or individual experiences, focuses on topics of self-care (taking care of oneself for physical and mental well-being), maintaining research activities and productivity, and leisure (social engagement and taking time for oneself), while conducting IKT research during the pandemic. At the meso level, the role of programmes and organizations explores whether and how institutions were able to adapt and continue research and/or partnerships during the pandemic. At the macro level, we discuss implications for policies to support IKT trainees and research, during and beyond emergency situations. Themes were identified that intersected across all levels, which included (i) equitable access to training and partnerships; (ii) capacity for reflexivity; (iii) embracing changing opportunities; and (iv) strengthening collaborative relationships. These intersecting themes represent ways of encouraging sustainable and equitable improvements towards establishing and maintaining collaborative health research approaches. This essay is a summary of our collective experiences and aims to provide suggestions on how organizations and universities can support future trainees conducting collaborative research. Thus, we hope to inform more equitable and sustainable collaborative health research approaches and training in the post-pandemic era.

**Supplementary Information:**

The online version contains supplementary material available at 10.1186/s12961-022-00900-8.

## Introduction

Collaborative health research approaches, such as integrated knowledge translation (IKT), have the potential to improve healthcare systems, services and outcomes worldwide by working with the people that these systems, services and outcomes directly affect, and with those who can implement evidence-based changes [1]. IKT is defined as “*a model of collaborative research, where researchers work with knowledge users who identify a problem and have the authority to implement the research recommendations*” [1]. The importance of collaborative research was evident during the first 2 years of the COVID-19 pandemic, with the urgent and substantial need for knowledge to be contextualized for different knowledge users, settings and research objectives [2, 3]. However, distancing measures and travel restrictions made it necessary to find new ways to develop relationships and maintain strong partnerships with knowledge users such as policy-makers, healthcare providers, patients and the public. IKT trainees who were conducting collaborative health research (i.e. undergraduates, graduate students, postdoctoral scholars) also had to adapt the way we work, learn and reflect on how our research fit within a rapidly changing health system.

The Integrated Knowledge Translation Research Network (IKTRN) was established in 2016 as an international network of researchers, knowledge users and trainees to advance the field of IKT. The IKTRN mission is “*to bring together knowledge users and researchers to advance the science and practice of IKT and train the next generation of IKT researchers*” [4]. The IKT trainees, including graduate and postdoctoral scholars, represent a subgroup of the IKTRN who engage in knowledge sharing, training and networking opportunities as part of the wider network. This group came together as part of a collaborative research initiative to explore our personal experiences and reflections during the first 2 years of the COVID-19 pandemic.

## The IKTRN trainee team

As a diverse team of 16 IKT trainees from across Canada and internationally (Ireland and Australia), we each bring a unique set of experiences and perspectives to this essay. We are from diverse backgrounds in terms of our years of IKT experience, levels of education and geographical locations, which helped to enrich our group discussions. However, we acknowledge that many perspectives are missing from the essay. All participants self-identified as female (gender), and the majority were Canadian. Participants were representative of the IKTRN trainee group at the time this work began, which included 30 members. In writing this essay, we each played multiple roles as data collectors, research participants, analysts and authors. Working together provided an avenue to learn from each other and strengthen relationships among IKTRN trainee members. All IKTRN trainees were invited to participate, and those who attended the group discussions were invited to contribute to this essay.

## Synthesizing our experiences

On 12 March 2021, the IKTRN Trainee Co-Chairs (SS and AC), in collaboration with the Executive Committee, hosted a virtual discussion entitled “Trainee Experiences during COVID-19” led by SS and AC to capture the collective experiences and perspectives of IKTRN trainees during the first year of the COVID-19 pandemic. Twenty-eight IKTRN trainees were invited, of which 11 attended (*N* = 9 Canada; *N* = 1 Ireland; *N* = 1 Australia). Trainees were randomly assigned into breakout groups of three to four participants. Three 25-minute virtual group discussions were conducted simultaneously using a semi-structured guide (developed by SS) (Additional file 1: Appendix S1). Discussions were audio- or video-recorded, transcribed verbatim (MG, BT, AC), and then de-identified. Discussion attendees could review their transcript prior to analysis. Attendees were also invited to submit written vignettes (*n* = 5) about their IKT trainee experiences during the COVID-19 pandemic. Vignettes were short (up to one page) reflections written by individual IKT trainees about their first year of the COVID-19 pandemic. These vignettes were submitted after attending the group discussions. Subsequent, ongoing group discussions also informed this essay, which thus reflects our experiences during the first 2 years of the COVID-19 pandemic.

Data analysis was informed by an interpretative phenomenological approach (IPA) [5–8] to describe and interpret the individual lived experiences of IKTRN trainees during the COVID-19 pandemic. Transcripts and vignettes were read independently by all authors, then three authors (CL, BW, MG) developed codebooks independently, and each codebook was reviewed by a “critical friend” (BT, TN, PM). Critical friends were responsible for reviewing the initial codebooks, asking questions, encouraging reflexivity and different ways of thinking, and flagging potential gaps. The codebooks were then merged and discussed prior to review by all authors. We acknowledge that this essay is based on our individual and collective experiences and insights as IKT trainees conducting collaborative health research. This collaborative reflection aims to provide insight into these experiences to inform other trainees about challenges and strategies to consider. We also acknowledge that although there was expanded focus on organizations and systems, our perspective is always as IKT trainees, and further work is needed to capture the ideas of our supervisors, research partners, universities and health system organizations.

## Experiences and reflections from IKT trainees

**Fig. 1:**
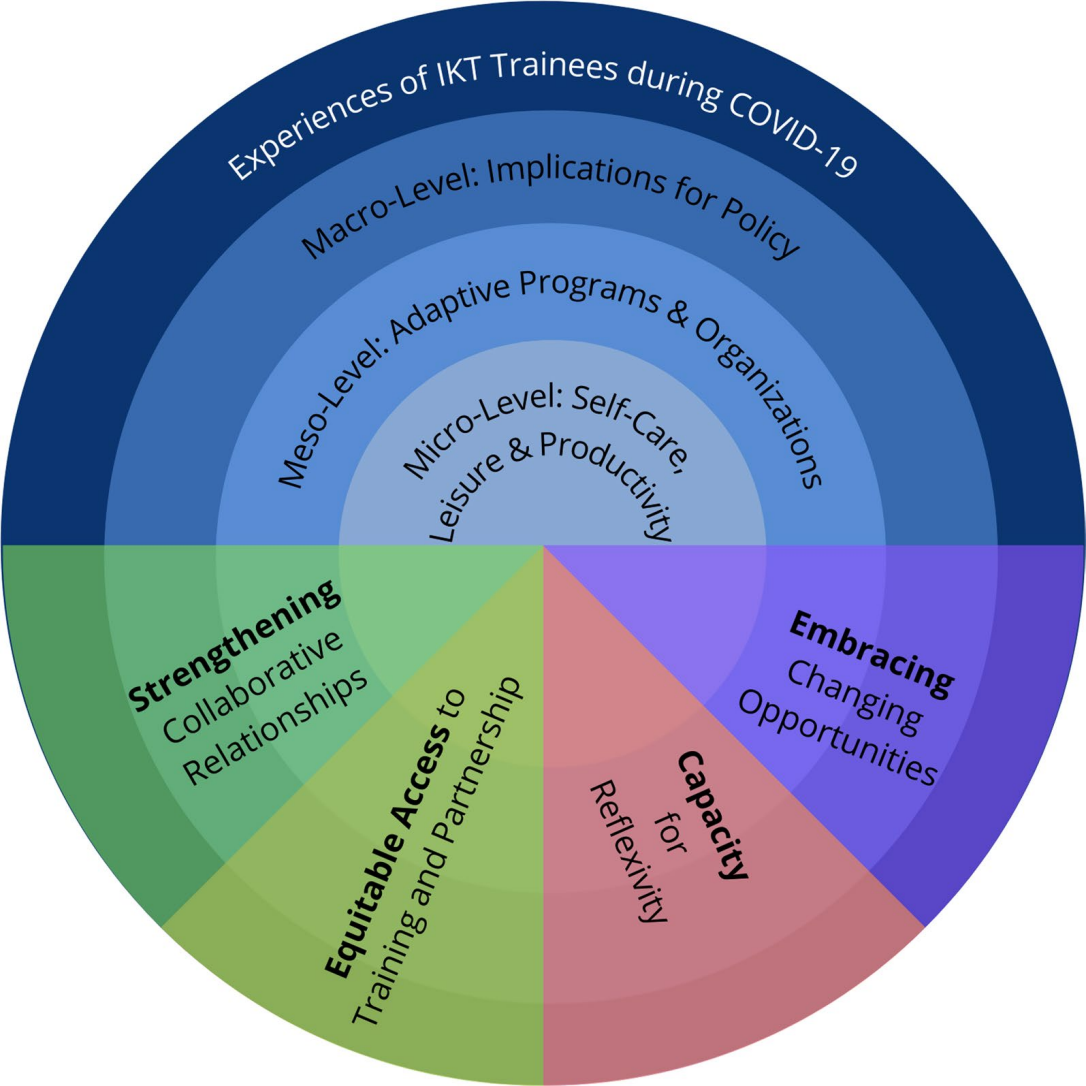
A visual representation of the experiences of IKT trainees during COVID-19 following the socio-ecological model. The socio-ecological model considers the complex interplay between individual, societal and structural factors

Our experiences and reflections as IKT trainees during the COVID-19 pandemic are highlighted below and presented in a visual summary (Fig. 1).

The socio-ecological model was used to frame our discussion and visual representation, as it considers the complex interplay between the micro (individual), meso (organizational) and macro (system) levels of influence and impact [9, 10]. This complex interplay of levels helps to deepen understanding of the range of factors that can affect people and how they live their lives [9]. This model was selected to emphasize the different ways in which IKT trainees were impacted, and to provide a conceptual way to frame our results, recognizing the interconnected nature of each of these levels. At the micro level, or level of individual experiences, we focus on topics of self-care (taking care of oneself for physical and mental well-being), maintaining research activities and productivity, and leisure (social engagement and taking time for oneself), while conducting IKT research during the pandemic. At the meso level, the role of organizations explores whether and how institutions were able to adapt, in order to continue supporting research and/or (maintain) partnerships during the pandemic. The macro level includes a discussion of implications for policies that support IKT trainees and research, including suggestions for universities and health system organizations.

Four areas were identified as “intersecting themes” that flowed across all three levels: (i) equitable access to training and partnerships, (ii) capacity for reflexivity, (iii) embracing changing opportunities and (iv) strengthening collaborative relationships.

In summary, we provide suggestions on how to support IKT trainees, and to inform equitable and sustainable collaborative health research practices in the post-pandemic era.

Table 1 summarizes suggestions on how to encourage sustainable and equitable changes in support of IKT trainees and future directions of research across levels (micro, meso, macro), as well as within the intersecting themes.

**Table 1:** Key actions to support IKT trainees and inform future topics in IKT research

Topics	Trainee quotes	Actions to support IKT trainees and inform future topics in IKT research
Micro level: self-care, leisure and occupation	“*While the pandemic has thrown a wrench in our way of life, it has also served as an opportunity for personal and professional growth in addressing its inherent challenges. Having said that, the absence of human connection and the spontaneity of ideas through face-to-face interactions is something that is immensely felt and missed*.” (V1)	*IKT trainees can* • Attend training and seek mentorship on ways of working in a hybrid (virtual and in-person) environment • Attend training on how to create a safe and inclusive space (virtual and in-person) • When needed, access available resources to support health and wellness, particularly mental health • Be attuned and adaptable to the needs of research partners *Trainees’ supervisors can* • Support trainees in accessing training opportunities to grow their skill set as an independent researcher. This can include funding to advance their training *Potential research topics* • Strengthening training in designing and facilitating hybrid research • Develop effective training on creating safe and inclusive spaces (virtually and in-person)
Meso level: adaptive programmes and organizations	“*My biggest concern is that it* [meeting online] *limited diversity because the people who were involved in these projects were people who had the resources to get online and you know, that tech knowledge of how to do a Zoom call. Even though we offered support, I worry that people who didn’t have confidence to use technology might not have been willing to be involved in that sort of research*.” (FG3)	*Programmes and organizations can* • Develop training and mentorship opportunities on how to create a safe and inclusive space (virtual and in-person). Identify other ways, specific to the organization, to ensure that technology does not limit engagement in research • Create opportunities for trainees to have informal discussions online to support networking, creativity and well-being • Support trainees to be involved in more sustainable relationships that adapt to health system needs through professional development opportunities (e.g. education session and workshops) • Supervisors should continually assess if their IKT research, and the research of their trainees, is meeting the needs of partners and community, while also supporting the trainee to graduate *Potential research topics* • Effective professional development content and/or formal training to support IKT trainees during times of health system crisis • Barriers and facilitators for IKT supervisors to rapidly developing and maintain research partnerships
Macro level: implications for policy	“*During the COVID-19 pandemic, new partnerships form to solve previously unforeseen problems. The pandemic raised urgent research questions that needed to be answered quickly, with solutions that could be implemented rapidly. I feel partnerships between researchers and stakeholders have been keenly supported during this time because both stakeholders and researchers alike recognized the potential to produce quality research that could be rapidly put into practice*.” (V5) “*I think we have learned that we can do things really quickly if we need to. Definitely, the projects that I have worked on that have been COVID-related have moved really quickly and engaged patients and clinicians*.” (FG3)	*Organizational and system level policy-makers can* • Develop organizational policies and guidelines so IKT trainees and their partners can access safe virtual spaces, and have the skills to create and sustain those safe spaces virtually and in-person • Create opportunities (i.e. webinars, seminars, workshops) for trainees to learn about how to connect with government officials and other decision-makers generally, so they are already established during times of crisis • Create national training programmes for all IKT trainees to engage in skill development, education and training to advance equitable and safe research collaboration *Potential research topics* • Effective training on rapidly developing partnerships with decision-makers that can adapt to system change • Best practices training on how to connect equity-deserving groups and decision-makers
Key intersecting themes		
Equitable access to training and partnership	Ensure IKT trainees and their knowledge user partners are provided with equitable, safe and inclusive opportunities and ongoing support to be involved in collaborative health research, such as through training, education, mentorship, organizational support, best-practice guidelines and opportunities to connect with policy- and decision-makers “*Learning and talking a bit more about creating safe spaces on virtual platforms for diverse groups you’re working with. especially those in rural communities who have to deal with issues around status disclosure and perhaps thoughts on consequences—around consequences of receiving care after the fact and participating in certain types of research?*” (FG2)	
Capacity for reflexivity	IKT researchers should promote reflective thinking among IKT trainees as a tool to identify and respond to personal, productivity or health system challenges, and thereby helping them to be better equipped to adapt to unforeseen events. IKT research can focus on effective reflexivity training, benefits and challenges of reflexive trainees, and how to increase trainee capacity to undertake this practice “*I have had the opportunity to reflect on my research journey and ask questions about how to leverage IKT in a time of crisis. … It is in this state of crisis that there is an opportunity to ask new questions, share experiences and perspectives, and co-create knowledge to overcome adversity and challenges. It is in this space that IKT will thrive and offer relevant and practical research that will have an impact*.” (V3) “*Postponing the start of my research has given me chance to self-reflect on my research progress. … It has taught me to be reflexive in my implementation plan and timeline, and to adapt to the unexpected circumstances rather than panic in the face of challenges when conducting research*.” (V2)	
Embracing changing opportunities	Supervisors can support IKT trainees to recognize and talk about changing health priorities, and provide opportunities to collaborate in different ways, such as how to engage with decision-makers and government officials, and how to increase global collaboration and learning. Research can be conducted about effective ways to provide training on how to embrace changing opportunities “*Social distancing has encouraged ‘out-of-the-box’ strategies for how we engage and partner with stakeholders in knowledge translation and implementation*.” (V1) “*I received additional opportunities to utilize my skills in co-production to support the development of research partnerships responding to the new realities of the pandemic*.” (V5)	
Strengthening collaborative relationships	Supervisors should continue training and mentoring trainees on the importance of developing equitable and sustainable relationships in a hybrid work environment. Future research could include the importance of and strategies for hybrid collaborative research, and its continuing role in global IKT research “*I was faced with the challenge of pivoting the direction of my community-based knowledge translation plan from conducting in-person to virtual focus groups, and learning to create safe digital spaces to facilitate didactic-intensive sessions to gather feedback from participants*.” (V2) “*The use of virtual workspaces/platforms for communication and interaction with team members and clients have assisted in breaking down geographical barriers to advance partnered research (e.g. IKT)*.” (V1)	

*IKT* integrated knowledge translation

### The micro level: self-care, occupation and leisure

The COVID-19 pandemic continues to have personal and professional impacts on graduate trainees [11, 12]. As IKT trainees, our experiences of conducting IKT research during the COVID-19 pandemic were categorized into the areas of self-care, productivity and leisure [13, 14]. As much of our work in IKT involves building relationships and co-creating knowledge, we recognize the need to be mindful of our own mental well-being, and that of our family and colleagues, in order to have capacity to build and sustain healthy relationships with our research partners. Anxiety, stress, fear of the unknown, feelings of being overwhelmed and a lack of control were common, as they were for other trainees not doing collaborative health research [11, 12]. Some IKT trainees, along with our colleagues, family and friends, were assigned unexpectedly to front-line work. “*I had this need to move away from my research and actually help out in healthcare*” (FG3). Some trainees experienced financial hardship and economic insecurity due to loss of employment, delayed graduation or changes in work schedules. Trainees tried to cope with some of these hardships by adapting their daily activities and trying to increase time spent in self-care, leisure or physical activity, and online social engagement. Some trainees experienced a pause in their personal and professional life that enabled them to engage in self-reflection about priorities, goals and objectives. This critical time of reflection allowed some trainees to reset, realign and re-envision new ways of working and building partnerships.

Trainees were also innovative in how they connected, communicated and collaborated with colleagues, knowledge users and other stakeholders using tools such as telephones, video conferencing software (i.e. Zoom), email, SMS (Short Message Service, i.e. texting) and online brainstorming tools (i.e. Mural). Trainees and knowledge users requested practical training from their institutions on how to use new tools. Even with efforts to engage online, some research activities had to stop due to facility closures, lack of participant recruitment or other issues. For example, one trainee discussed a project co-led by lived experience advisors, which relied on physician recruitment by the health system partner. The trainee described not wanting to disappoint the team, but eventually having to cancel the project. “*I don’t want to let them down when the project doesn’t work, but *[I’m]* still trying to make sure that they *[lived experience advisors]* can still contribute in the ways that they want*” (FG1). Trainees at the analysis stage of their work experienced less disruption. “*Because we got the data collection done before the COVID really started, or really hit hard, there was stuff the research team could keep doing*” (FG1).

Individually, IKT trainees discussed how we should attend training and seek mentorship on ways of working in a hybrid (virtual and in-person) environment and how to create safe and inclusive spaces as the global pandemic unfolds and beyond. When needed, IKT trainees should access available resources to support health and wellness, particularly to support their mental health. More research is needed on how to strengthen IKT training in a hybrid environment, including how to effectively develop safe spaces, acknowledging the role of power dynamics and trust in online or hybrid meetings. More research is also needed on developing and maintaining research partnerships in times of crisis, including understanding barriers and facilitators from the perspective of each partner.

### The meso level: adaptive programmes and organizations

The meso level focuses on the way we, as IKT trainees, work and function within our programmes and organizations (i.e. universities, research institutes, hospitals), and how we develop and maintain partnerships with colleagues and other stakeholders throughout our research. During the pandemic, organizations had to quickly adapt to fluctuating public health priorities and policies. Physical distancing measures resulted in cancellation of face-to-face meetings. Only those who had capacity to remain involved and access to resources were able to transition to virtual video format. “*I know one group that I have been working closely *[with]*, they’re actually shipping out iPads to communities in Ontario in order to participate in their work… but that certainly takes dollars and isn’t available to everyone and still doesn’t address the Internet aspect of it*” (FG2).

In addition, IKT trainees reported fewer opportunities to engage and connect with other trainees, colleagues, supervisors or mentors. Virtual platforms limited the spontaneous, informal conversations that often lead to creativity, ideas and new opportunities. Some trainees shared that the dynamics of how we interact as humans has been dramatically altered through virtual meetings. This has significant implications for trainees and collaborative research partnerships, which are focused on developing and nurturing trusting relationships. Despite these challenges, many trainees received additional support from supervisors and mentors, including resources, timeline flexibility and new opportunities to collaborate.

As IKT trainees, we encourage supervisors and academic institutions to promote opportunities for informal “chats” in person and online to help build the individual relationships essential to IKT, while also supporting the well-being of trainees and partners [15]. “*A lot of those kind of moments between formal things have been lost… opportunities to quickly check in with someone or have like a normal interaction and then kinda get to business and talk about what the topic for that meeting was that day … I do kinda miss having more informal dialogue with people… that whole kind of part of how we interact has really changed*” (FG2). We also suggest organizations account for the additional time and adaptations needed by supervisors to ensure their own IKT research, and that of their trainees, continues to meet the needs of partners and community, while also supporting trainees to graduate.

Redeployment of staff and a reduced workforce led to changes in workflow and capacity of trainees. Decisions on whether research should continue, pause or be cancelled completely decreased both employment and training opportunities for IKT trainees. For example, engagement with patients abruptly stopped for many research programmes across Canada, while others changed the way they engaged with patients [16–19]. Ultimately, there were limited resources and reduced capacity to engage in collaborative and partnership research. As highlighted by one trainee working on a project with one of the populations indigenous to Canada: “*The First Nations were being hit quite hard with COVID, and no one wanted to be—it’s not a priority to be engaged in research when you have people in your communities, and your family members being sick and dying. Like, that’s, research is not a priority*” (FG1). Encouragement of ongoing collaboration rather than project-specific partnerships may build a platform for more sustainable relationships, allowing the work to adapt to health system needs, while still valuing project-specific partnerships that meet trainee needs [17, 18].

Despite the challenges presented by the pandemic, new opportunities and spaces emerged where collaborative research could flourish. The need for research partnerships during COVID-19 exploded with increased opportunities for rapid action and population-level impact. Although there were many new partnerships forming, there is a need to understand how to sustain new and existing national and international partnerships to ensure these partnerships remains at the forefront of research.

### The macro level: implications of policy

During the pandemic, many of us saw the growth and recognition of collaborative research within the scientific community through self-reflection. “*I feel like it’s much better-positioned me, and all of us, especially everyone in this field. We’ve watched what happens now when everybody gets on board and pays attention to the latest and greatest science. And how quickly we can get things done. And I’m hoping that that, combined with online collaborations like this, will lead to much faster change*” (FG3).

At an organizational policy level, guidelines or assistance in following organizational direction for remote work may help trainees and research partners feel supported during times of crisis, which may or may not currently be in place. We realize the rapid and dynamic environment the pandemic provoked; however, too much change, too fast, without ongoing support can have negative implications on mental health, professional advancement, and relationships with research partners. To support ongoing learning, we suggest that organizational- and system-level decision-makers create more opportunities for trainees to connect with and learn from government officials and other decision-makers throughout the policy development and enactment processes in general.

#### Intersecting themes at the micro, meso and macro levels

Four key intersecting themes were identified in the micro, meso and macro levels, including (i) equitable access to training and partnerships; (ii) capacity for reflexivity; (ii) embracing changing opportunities; and (iv) strengthening collaborative relationships. These themes were identified at all levels and begin to indicate potential areas for action. Our aim here is to elaborate on how these intersecting themes work together, rather than exploring each individually, as there is considerable overlap between the themes.

To support equitable access to training and partnership opportunities, organizations need to provide resources, technology and training (for both trainees and research partners) in virtual platforms and hybrid engagement. The cost of reliable technology and high-speed Internet/connectivity is not readily accessible to all. Those with connectivity issues in rural areas or without access to reliable computers require tools to enable participation. As IKT trainees, we also need training on how to create and maintain a safe space online, which acknowledges the challenges of building trust and addressing power dynamics in an online or hybrid meeting. We encourage creative strategies to engage individuals who are not able or comfortable with online interaction. To ensure equitable access to partnerships, the field of IKT also needs to do more to ensure individuals and communities are provided the opportunity and ongoing support to become partners in the research.

For some of us, the pandemic provided an opportunity to take a step back and reflect on our personal values, and the impact of our work. Others found that this capacity for reflection also meant more time to worry, while some had no time to think. Reflexivity in collaborative health research practices is a skill that trainees need to embrace in order to respond to and adapt to any unforeseen challenges or contextual factors.

Opportunities changed quickly throughout the pandemic. There were new online learning opportunities that were not previously accessible, yet limited opportunities (i.e. lack of technology to support one-to-one conversations in online group settings) for personal connections while attending those online sessions. Some trainees were overwhelmed with homeschooling their children and remote working, yet appreciated the chance to spend more time together as a family. The increasing access to global collaborations broadened networking opportunities and the chance to work with new people, organizations, projects and research interests, and thus needs to be continued outside the pandemic.

## Conclusion

The COVID-19 pandemic had a significant impact on IKT trainees at the micro, meso and macro levels. IKT researchers must advocate for and promote training opportunities to IKT trainees and research partners on how to develop and sustain equitable collaborative research partnerships that meet and adapt to the needs of knowledge users. Trainees need to be better equipped to embrace hybrid models of collaborating and working both in person and virtually. We can work with our supervisors, mentors and organizations to facilitate access to resources and technology for our partners and ourselves. The pandemic has heightened the need to value our own health and well-being in order to have the capacity to be adaptable to the needs of our research partners.

Universities and health system organizations can be adaptive to changing priorities, while also supporting the health and well-being of individuals within their organization, particularly trainees. Even though the pandemic has demonstrated that partnerships with knowledge users can pivot to a virtual platform and form rapidly, we acknowledge the value of maintaining and nurturing existing partnerships through both face-to-face and virtual formats. With sustained relationships, it may be more feasible for universities and organizations to adapt to changing health system needs, and IKT trainees are a key part of this sustainability.

## Supplementary Information

**Additional file 1.** Breakout session facilitation guide.

## Data Availability

Not applicable.

## Abbreviations

IKT: Integrated knowledge translation
IKTRN: Integrated Knowledge Translation Research Network
IPA: Interpretative phenomenological approach
