# Supplementary material for: Building capacity for integrated knowledge translation: a description of what we can learn from trainees’ experiences during the COVID-19 pandemic
Source: Health Res Policy Syst. 2022 Sep 15;20:100. doi: 10.1186/s12961-022-00900-8 (PMC9479415; doi:10.1186/s12961-022-00900-8)
Supplement: Supplementary file 1 — Additional file 1. Breakout session facilitation guide. [file 12961_2022_900_MOESM1_ESM.docx]

**Breakout Session Facilitation Guide**

**March 12, 2021**

| **Impact of Covid-19 Pandemic on IKT Research Trainee Experience** | |
| --- | --- |
| INTRODUCTIONS (~ 5 min) | |
| - Share a fun fact about yourself and your research interests | |
| COVID-19 IMPACT: (~ 10 min) | |
| Personal Impact | - How have you been impacted personally by the pandemic- share your thoughts, feelings, insights, perspectives - Was there a direct impact on your mental health- ie. motivation, stress, anxiety, fatigue, fears, daily function, energy levels, productivity, scheduling, time, sleep etc) - What strategies and supports have been helpful? |
| Research Impact | - What were the barriers and challenges to doing research - Any delays, changes or adaptations to your research approach or process - What was the impact on partners and partnership development |
| New opportunities | - Access to new opportunities or doors that opened - Virtual learning or networking - Connecting internationally or globally |
| Future impact on IKT research | - Consider how the shift to virtual may change how we do research and the type of research, context/environment |
| IKTRN Trainee Network – Future Opportunities (~ 5 min) | |
| Consider how to optimize the IKT Trainee experience going forward? | - Workshops, webinars, guest speakers - Peer to peer learning; co-lead information/education sessions - Experiential learning (grants, publication) - Formal training opportunities (ie. Fellowship or IKT trainee modules) |
| SUMMARIZE KEY POINTS (5 min) | |
|  | - REPORT BACK TO LARGE GROUP |
